# Targeted Auger electron-emitter therapy: Radiochemical approaches for thallium-201 radiopharmaceuticals

**DOI:** 10.1016/j.nucmedbio.2021.03.012

**Published:** 2021-04-17

**Authors:** Alex Rigby, Julia E. Blower, Philip J. Blower, Samantha Y.A. Terry, Vincenzo Abbate

**Affiliations:** a King’s College London, School of Biomedical Engineering and Imaging Sciences, 4th Floor Lambeth Wing, St Thomas’ Hospital, London SE1 7EH, United Kingdom; b King’s College London, School of Population Health and Environmental Sciences, Analytical, Environmental and Forensic Sciences, Franklin-Wilkins Building, Stamford Street, London SE1 9NH, United Kingdom

**Keywords:** Thallium-201, Auger electrons, Chelator, Molecular radionuclide therapy, Oxidation

## Abstract

**Introduction:**

Thallium-201 is a radionuclide that has previously been used clinically for myocardial perfusion scintigraphy. Although in this role it has now been largely replaced by technetium-99 m radiopharmaceuticals, thallium-201 remains attractive in the context of molecular radionuclide therapy for cancer micrometastases or single circulating tumour cells. This is due to its Auger electron (AE) emissions, which are amongst the highest in total energy and number per decay for AE-emitters. Currently, chemical platforms to achieve this potential through developing thallium-201-labelled targeted radiopharmaceuticals are not available. Here, we describe convenient methods to oxidise [^201^Tl]Tl(I) to chelatable [^201^Tl]Tl(III) and identify challenges in stable chelation of thallium to support future synthesis of effective [^201^Tl]-labelled radiopharmaceuticals.

**Methods:**

A plasmid pBR322 assay was carried out to determine the DNA damaging properties of [^201^Tl]Tl(III). A range of oxidising agents (ozone, oxygen, hydrogen peroxide, chloramine-T, iodogen, iodobeads, trichloroisocyanuric acid) and conditions (acidity, temperature)were assessed using thin layer chromatography. Chelators EDTA, DTPA and DOTA were investigated for their [^201^Tl]Tl(III) radiolabelling efficacy and complex stability.

**Results:**

Isolated plasmid studies demonstrated that [^201^Tl]Tl(III) can induce single and double-stranded DNA breaks. Iodo-beads, iodogen and trichloroisocyanuric acid enabled more than 95% conversion from [^201^Tl]Tl (I) to [^201^Tl]Tl(III) under conditions compatible with future biomolecule radiolabelling (mild pH, room temperature and post-oxidation removal of oxidising agent). Although chelation of [^201^Tl]Tl(III) was possible with EDTA, DTPA and DOTA, only radiolabeled DOTA showed good stability in serum.

**Conclusions:**

Decay of [^201^Tl]Tl(III) in proximity to DNA causes DNA damage. Iodobeads provide a simple, mild method to convert thallium-201 from a 1+ to 3+ oxidation state and [^201^Tl]Tl(III) can be chelated by DOTA with moderate stability. Of the well-established chelators evaluated, DOTA is most promising for future molecular radionuclide therapy using thallium-201; nevertheless, a new generation of chelating agents offering resistance to reduction and dissociation of [^201^Tl]Tl(III) complexes is required.

## 1 Introduction

Since first being described by Lise Meitner and Pierre Auger in the 1920s, Auger electrons (AEs) have been investigated for use in molecular radionuclide therapy (MRT). AEs are a product of radionuclide decay, typically via electron capture or internal conversion, occurring in large numbers (4.7–36.9 per disintegration) and at low energies (<25 keV) [1]. However, this energy is deposited across a small distance (<0.5 μm), leading to higher linear energy transfer than radiotherapies involving, for example, beta particles with energies up to 2 MeV, where energy is deposited over 0.1–10 mm. For reference, alpha particle-emitters deposit their energy over 40–80 μm [2]. AE-emitters could therefore permit highly targeted therapies, capable of extreme radiotoxicity, even in micrometastases and single circulating tumour cells, but only if they can be delivered to certain targets such as the cell nucleus or membrane [3,4]. AE emissions accompany the decay of many radionuclides used in medical imaging, including ^111^In, ^67^Ga, ^99m^Tc, ^64^Cu and ^201^T1, thereby allowing therapeutic radionuclides to be tracked to their biological target using single photon emission computed tomography (SPECT) or positron emission tomography (PET; in the case of ^64^Cu) imaging.

The majority of AE-emitting MRT studies have utilised indium-111 and iodine-125 [5–14]. Previous molecules, such as [^125^I]I-IUdR, effectively killed cancer cells by covalently binding DNA [9]. Others have successfully used antibodies incorporating nuclear localisation sequences to amplify the effectiveness of AEs [7]. Recently, the therapeutic efficacy of an iodine-123-labelled poly(ADP-ribose) polymerase 1 (PARP1) inhibitor ([^123^I]I-MAPi) in glioblastoma models was presented [15]. Generally, however, despite excellent preclinical results, translation of AE to the clinic has met with limited therapeutic impact due to their inability to deliver a lethal dose to the tumour. One example is [^111^In]In-Octreotide, which so far has come closest to clinical translation [8]. Future AE-emitting MRT may be more successful if a more potent AE-emitting radionuclide was used that emitted many AEs per decay, such as thallium-201 or platinum-195 m.

Thallium-201 (t_1/2_ = 73 h, [^201^Tl]Tl) has been used in medical imaging since the 1970s for myocardial perfusion SPECT imaging [16]. However, it has fallen out of favour since the development of technetium-99m-based agents, such as sestamibi and tetrofosmin, due to its long physical half-life (73 h) and consequent high absorbed radiation dose compared to technetium-99m (6 h) as well as the ready availability of technetium-99m from a generator. In 2005, it was demonstrated that clinical myocardial blood flow scans with thallium-201 led to genotoxicity in lymphocytes at day 3 after administration [17], highlighting the potential of healthy tissue toxicity from thallium-201. Similarly, intravenous injection of thallium-201 led to high testis uptake and toxicity in mice [18,19].

Thallium-201 releases an average of 36.9 AEs per decay at an average total energy of 15.3 keV per decay; higher than for gallium-67 (4.7 AEs and 6.3 keV), which we have investigated for MRT recently [20,21], indium-111 (14.7 AEs and 6.8 keV) and iodine-123 (14.9 AEs and 7.4 keV) (Table 1; [1]). Indeed, thallium-201 resembles iodine-125 (24.9 AEs and 12.1 keV) in its electron-emitting properties, although its half-life is more favourable for clinical radiopharmaceutical use than iodine-125.

There are few published therapeutic studies involving thallium-201. Early studies in the 1980s highlighted toxicity of thallium-201 in V79 Chinese hamster lung fibroblasts [22]. Others have relied on *in silico* simulations. For example, Monte Carlo computational methods were used to accurately model the radiation dose from thallium-201 at target volumes of <1 μm in diameter by taking into account the contribution from AEs [23–26]. More recently, Geant4-DNA, another Monte Carlo simulation toolkit, demonstrated the theoretical number of single and double strand breaks that could be produced by AE-emitters on the DNA scale; thallium-201 was amongst the most effective in causing DNA damage [27].

Thallium-201 radiobiological studies have been compounded due to the difficulty of synthesising a thallium-201 -labelled radiopharmaceutical. Whereas putative ^201^T1-labelled drugs like bleomycin and vancomycin have been assessed as imaging agents [28,29], a bifunctional chelator that forms a stable complex with thallium-201 still needs to be developed to accurately deliver the radionuclide to a tumour. lt is expected that thallium-201 needs to be converted from its commercially available 1 + to a 3 + oxidation state, which is more amenable to complexation by multi-dentate ligands. However, oxidation methods suggested to date require harsh conditions (such as high temperature and concentrated acid) incompatible with biomolecules [30]. Moreover, reported stability studies with DTPA as chelator have been inconsistent or inconclusive [31,32], justifying additional investigations to identify ligands suitable for use in MRT with [^201^Tl]Tl(III).

This work aims to (i) develop a mild, biomolecule-compatible method for oxidising [^201^Tl]Tl(I) to [^201^Tl]Tl(III), (ii) determine the DNA damaging potential of [^201^Tl]Tl(III), and (iii) assess commonly used, commercially available chelators for [^201^Tl]Tl(III).

## 2 Materials and methods

**Fig. 1:**
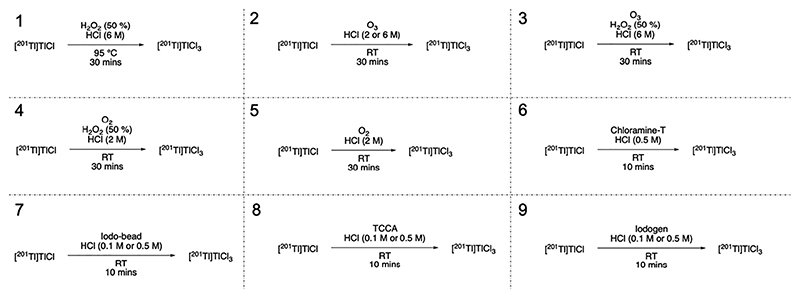
Oxidation methods used to convert [^201^Tl]TlCl to [^201^Tl]TlCl_3_.

Unless stated otherwise, chemicals and solvents were purchased from commercial suppliers (Merck, Fisher Scientific, CheMatech). [^201^Tl]TlCl in saline was purchased from Curium Pharma, UK, and converted to [^201^Tl]TlCl_3_ by one of nine methods described below and summarised in Fig. 1.

### 2.1 Oxidation method 1 - HCl (6 M), H_2_O_2_, and 95 °C

HCl (6 M, 300 µL) was added to [^201^Tl]TlCl (11.2 MBq, 200 µL). Hydrogen peroxide (50% in water, 100 µL) was then added and the solution vortexed for 10 s and placed in a pre-heated heat block at 95 °C for 30 min.

### 2.2 Oxidation method 2 - HCl (2 or 6 M) and ozone

HCl (2 or 6 M, 200 µL) was added to [^201^Tl]TlCl (11.2 MBq, 200 µL). Ozone produced from medical grade oxygen via an ozone generator (1KNT-24 from Enaly, China) was bubbled through the radioactive solution via a glass pipette for 30 min.

### 2.3 Oxidation method 3 - HCl (6 M), H_2_O_2_ and ozone

HCl (6 M, 200 µL) and hydrogen peroxide (50% in water, 50 µL) were added to [^201^Tl]TlCl (16.8 MBq, 300 µL). Ozone was used as in method 2.

### 2.4 Oxidation method 4 - HCl (2 M), H_2_O_2_ and oxygen

HCl (2 M, 200 µL) and hydrogen peroxide (50% in water, 50 µL) were added to [^201^Tl]TlCl (11.2 MBq, 200 µL). Oxygen, directly fromamedical grade oxygen cylinder, was bubbled through the radioactive solution via a glass pipette for 30 min.

### 2.5 Oxidation method 5 - HCl (2 M) and oxygen

HCl (2 M, 200 µL) was added to [^201^Tl]TlCl (11.2 MBq, 200 µL). Oxygen was used as in method 4.

### 2.6 Oxidation method 6 - chloramine-T

Chloramine-T (N-chlorotoluenesulfonamide; 0.1 mg – 10 mg) in water was added to a minicentrifuge tube. [^201^Tl]TlCl (5.2 MBq, 100 µL) was then added and the mixture was agitated for 10 min. Once dissolved, HCl (0.5 M, 100 µL) was added. A white solid precipitated from the solution. The solution was then agitated for 2 min, centrifuged for 30 s using a mini benchtop centrifuge to pellet the solid. The supernatant, containing [^201^Tl]Tl(III), was then added to a clean flask. This was then used for the chelator studies.

A non-radioactive version of the reaction in method 6 was performed and the white solid precipitate was analysed using proton NMR. NMR spectra were recorded on a Bruker Ultrashield 400WB PLUS 9.4 T spectrometer (^1^H NMR at 400 MHz). All chemical shifts were referenced to residual solvent peaks and are quoted in ppm. ^1^H NMR (400 MHz, Chloroform-d) δ 7.79 (d, *J* = 8.4 Hz, 2H, Ar-H_a_), 7.29 (d,*J* = 8.0 Hz, 2H, Ar-H_b_), 2.41 (s, 3H, Me).

Varying amount of chloramine-T, dissolved in water, was added to clean reaction flasks 10 ng - 0.1 mg. [^201^Tl]TlCl (1 MBq, 25 µL) was then added to the tubes containing the chloramine-T solution, followed by HCl (0.1 M, 0.5 M or no acid added, 2.5 µL), vortexed and pipetted into a flask.

### 2.7 Oxidation method 7 - iodo-bead

[^201^Tl]TlCl (0.5 MBq, 100 µL) was added to one iodo-bead (Thermo Fisher). HCl (0.1 M or 0.5 M, 10 µL) was then added to the reaction and vortexed for 10 s.

### 2.8 Oxidation methods 8 and 9 - trichloroisocyanuric acid (TCCA) and iodogen

In direct comparative studies, 10 ng - 0.1 mg iodogen and TCCA, both dissolved in chloroform and left in a fume hood overnight for the chloroform to evaporate, were added to clean reaction flasks. [^201^Tl]TlCl (1 MBq, 25 µL) was then added to the pre-coated tubes, followed by HCl (0.1 M, 0.5 M or no acid added, 2.5 µL), vortexed and pipetted into a flask.

### 2.9 Radiolabelling chelators

[^201^Tl]TlCl_3_ (40 µL, 3 MBq), produced by Chloramine-T method 6, was added to Eppendorf tubes containing 1 mg/mL ethylenediaminetetraacetic acid (EDTA) (0.34 μmol), diethylenetriaminepentaacetic acid (DTPA) (0.25 μmol) or 1,4,7,10-tetraazacyclododecanetetraacetic acid (DOTA) (0.25 μmol) in ammonium acetate buffer (0.25 M, pH 5,100 µL). The mixture was vortexed and agitated for 5 (EDTA and DTPA) or 60 min (DOTA) at room temperature and analysed using thin layer chromatography (as described below) to determine radiochemical yield.

### 2.10 Stability of[^201^Tl]Tl(III)-EDTA, [^201^Tl]Tl(III)-DTPA and [^201^Tl]Tl(III)- DOTA

Serum (300 µL), obtained from a healthy male volunteer, was added to an Eppendorf tube, followed by the relevant [^201^Tl]Tl(III)-chelator complex (200–300 kBq, 20–30 µL). This was then vortexed and incubated at 37 °C for up to 144 h. Similar stability studies were also carried out for [^201^Tl]Tl(III)-DOTA incubated in cell culture medium (RPMI- 1640) supplemented with 10% foetal bovine serum, 2 mM L-glutamine, and penicillin/streptomycin or in 0.25 M ammonium acetate buffer (pH 5) at 37 °C for up to 144 h.

### 2.11 Thin layer chromatography

Oxidation from [^201^Tl]Tl(I) to [^201^Tl]Tl(III) was analysed by instant thin layer chromatography (iTLC) with acetone as the mobile phase and silica gel IT1C strips (iTLC-SG) as the stationary phase, giving good separation between [^201^Tl]Tl(I) (R_f_ = 0) and [^201^Tl]Tl(III) (R_f_ = 1; Fig. S1).

Chelation of [^201^Tl]Tl(III) was analysed with reverse phase T1C plates (TLC Silica Gel 60 RP-18 F254s MS-grade) as the stationary phase and acetonitrile (30%)/water as the mobile phase, giving good separation between [^201^Tl]Tl(I) (R_f_ = 0) or [^201^Tl]Tl(III) (R_f_ = 0) and [^201^Tl]Tl(III)- EDTA/DTPA/DOTA (R_f_ =1). T1C plates were imaged using a Cyclone Plus Phosphor Imager (PerkinElmer, Inc. USA) or a LabLogic Radio TLC scanner (Sheffield, UK).

### 2.12 Plasmid DNA damage

pBR322 DNA plasmid (New England Biolabs, UK) in PBS (100 ng, 20 µL) was incubated with 0.5 MBq (8 µL) [^201^Tl]TlCl_3_ for upto144 h. [^201^Tl] TlCl_3_, originally formed using chloramine-T (method 6), was neutralised with Na_2_CO_3_ (0.1 M). Controls included untreated plasmid in PBS and equivalent amounts of non-radioactive [^nat^Tl]TlCl_3_ (Sigma). After treatment, plasmid (50 ng in PBS) was mixed with 6× loading dye (16 µL total volume), loaded onto a 0.8% agarose gel containing 10 µL GelRed Nucleic Acid stain (Biotium, USA) and run at 100 V (400 mA, 50 W) for 40 min. Gels, imaged using a GelDoc-ItTS2 310 Imager system (BioRad, UK) coupled with a Benchtop UV transilluminator (UVP) and GelCam 310, were analysed by ImageJ, measuring supercoiled (intact DNA), relaxed circular (single strand breaks) and linear band (double strand breaks) percentages within a lane (n = 3–12) [33].

### 2.13 Statistical analysis

Plasmid electrophoresis results are shown as mean percentage of total DNA, i.e. supercoiled + relaxed + linear topologies, ± standard deviation. Two-way ANOVA statistical analyses were carried out using Tukey’s multiple comparisons test in GraphPad Prism 7.0c. *P* < 0.05 was deemed significant.

## 3 Results

### 3.1 Ozone and oxygen oxidation

Table 2 summarises radiochemical yields obtained. Radiochemical yields of method 1 were 98 ± 2% (Fig. S2) whereas oxidation method 2 using a low concentration of HCl (2 M) with ozone yielded litT1e to no [^201^Tl]Tl(III) (3 ± 2%) (Fig. S3). Increasing the concentration to 6 M HCl slighT1y improved radiochemical yields to 12 ± 3% (Fig. S4). In a further attempt to oxidise thallium-201 at room temperature, oxidation method 3, which used a mixture of ozone, hydrogen peroxide and 6 M HCl was evaluated. This produced [^201^Tl]Tl(III) after 30 min (95 ± 5%, Fig. S5). Using oxygen instead of ozone and decreasing the pH to 2 M still yielded quantitative conversion from [^201^Tl]Tl(I) to [^201^Tl]Tl(III) (99 ± 1%; method 4). The further removal of hydrogen peroxide from the reaction still led to [^201^Tl]Tl(III) yield of 94 ± 6% (method 5).

### 3.2 Chloramine-T oxidation and NMR

lTLC analysis of the supernatant containing [^201^Tl]Tl(III) showed that quantitative yields were obtained using 10 mg Chloramine-T (99 ±1%; method 6), and that the presence of acid was required (Fig. S6). When 0.1 M HCl was used, a mixture of [^201^Tl]Tl(I) and [^201^Tl]Tl(III) was observed with 100 ng - 0.1 mg; no oxidation occurred at 10 ng. However, with the use of 0.5 M HCl, a yield of >99% for [^201^Tl]Tl(III) was observed at all concentrations at or above 0.001 mg. NMR spectroscopy showed the white precipitate to be p-toluenesulfonamide, an expected byproduct of the chloramine-T oxidation [34] (Fig. S7).

### 3.3 Solid phase oxidants

lodo-beads, incubated with [^201^Tl]TlCl in the presence of 0.5 M HCl, led to the formation of [^201^Tl]Tl(III) in 99 ± 1% yield (method 7A, Fig. S8). Using 0.1 M HCl instead decreased the radiochemical yield of [^201^Tl]Tl(III) to 62 ± 8% (method 7B, Fig. S6). TCCA alone (10 ng – 0.1 mg), without HCl, produced 88–90% conversion to [^201^Tl]Tl(III) within 10 min at room temperature (Fig. S6). Upon addition of 0.5 M HCl, full conversion (99 ± 1%) was observed between 10 ng – 0.1 mg TCCA (method 8A, Fig. S6). When using 0.1 mg iodogen without HCl, a radiochemical yield of [^201^Tl]Tl(III) at 74 ± 3% was observed (Fig. S6). Upon the addition of 0.1 M HCl using 0.001–0.01 mg iodogen led to a 95 ± 3% radiochemical yield, which further increased to 99% ± 1% when using 0.5 M HCl (Fig. S6).

### 3.4 DNA damage assessment

**Fig. 2:**
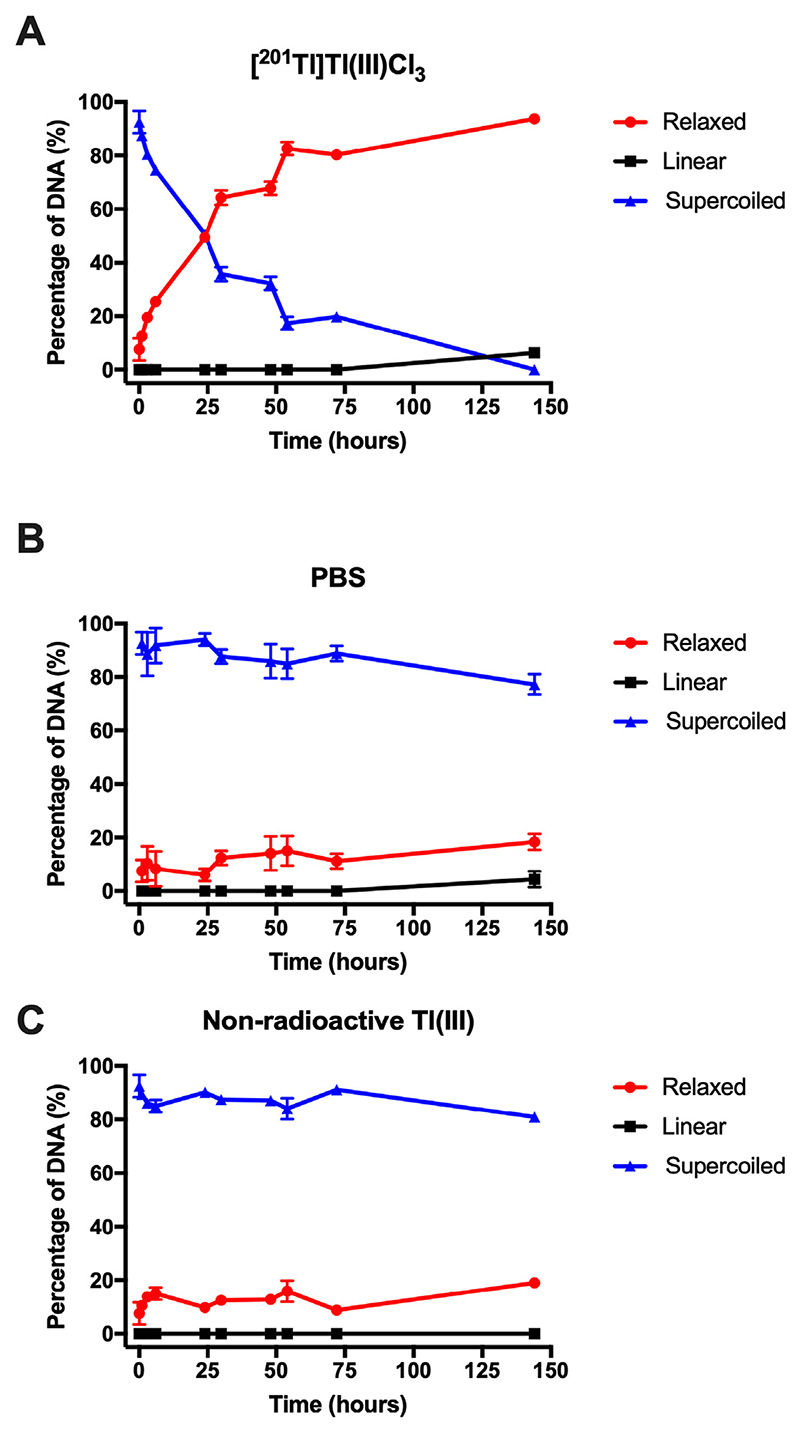
The percentage of DNA damage when plasmid DNA was incubated with [^201^Tl]Tl(IiI) Cl_3_, PBS (B), or non-radioactive T1(III) (C). Blue line = supercoiled, undamaged DNA, red line = DNA in relaxed form after a single strand break, black line = DNA in the linear form after a double strand break (n = 3–12). (For interpretation of the references to colour in this figure legend, the reader is referred to the web version of this article.)

For plasmid DNA incubated with 0.5 MBq [^201^Tl]Tl(III), increasing the incubation time decreased the percentage of supercoiled DNA from 88 ± 1% to 51 ± 2% at 1 and 24 h, respectively (Figs. 2, S9). The presence of relaxed DNA increased from 12 ± 1% at 1 h to 49 ± 2% at 24 h, whereas linear DNA was first detectable (6.27 ± 0.15%) at 144 h. ln all studies, negative controls consisting of the addition of PBS or non-radioactive [^nat^Tl]Tl(III) to the plasmid did not show evidence of damage over the corresponding timeframe within the errors associated with the measurement (Fig. 2; *p* = 0.22).

### 3.5 Chelation

**Fig. 3:**
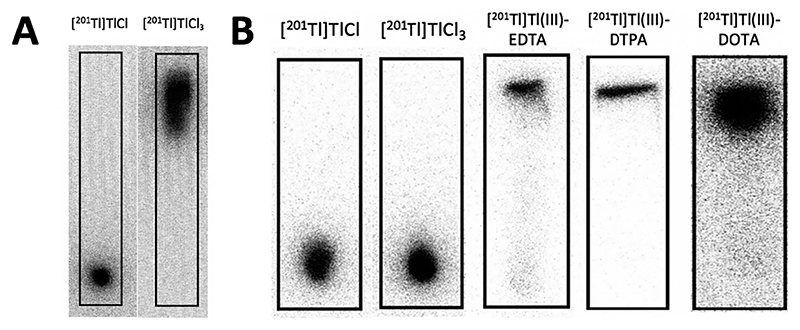
A Representative phosphor images of iTLCs of oxidation method 5 - HCl (2M) and oxygen. Solid phase = ITLC-SG, and mobile phase = acetone. R_f_0 = [^201^T1T1(I), R_f_1 = [^201^Tl]Tl(III). B Representative phosphor images of reverse phase TLCs of [^201^Tl]TlCl, [^201^Tl]TlCl3, [^201^Tl]Tl(III)-EDTA, [^201^Tl]Tl(III)-DTPAand [^201^Tl]Tl(III)-DOTA; [^201^Tl]Tl(I) and [^201^Tl]Tl(III) remain atthe origin (Rf = 0) while [^201^Tl]Tl(III) chelates migrate with the solvent front (Rf = 1).

[^201^Tl]Tl(III) formed using chloramine-T (method 6) was reacted with chelators EDTA, DTPA and DOTA. Reverse phase T1C plates, using acetonitrile (30%):water as the mobile phase, gave excellent separation of [^201^Tl]Tl(III)-EDTA, [^201^Tl]Tl(III)-DTPA and [^201^Tl]Tl(III)-DOTA from uncomplexed thallium-201 and showed >95% radiolabelling yield in all cases (n = 3; Fig. 3B).

### 3.6 Serum stability

**Fig. 4:**
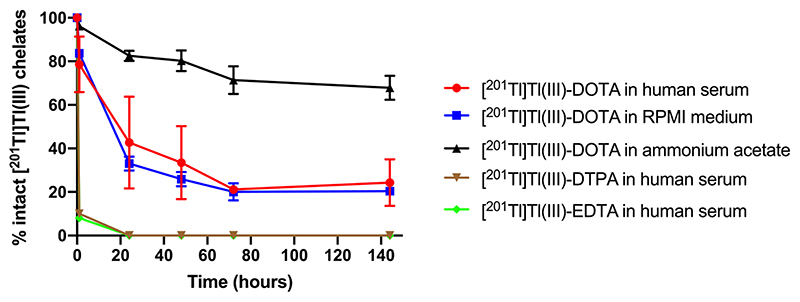
Stability of[^20^’Tl]Tl(III)-EDTA,[^20^’Tl]Tl(III)-DTPAand[^20^’Tl]Tl(III)-DOTAin humanserumandof[^20^’Tl]Tl(III)-DOTAmRPMl-1640mediumand0.25Mammoniumacetate(pH5)at 37 °C over 144 h. Values are average ± standard deviation (n = 3).

After 1 h in serum, of the [^201^Tl]Tl(III)-DTPA and [^201^Tl]Tl(III)-EDTA formed after the initial complexation, only 9 ± 2% remained; the complexes had completely dissociated by 24 h (Fig. 4). ln comparison, [^201^Tl]Tl(III)-DOTA dissociated at a slower rate with 78 ± 12% of the complex remaining at 1 h, and 24 ± 13% of [^201^Tl]Tl(III)-DOTA still intact at 144 h. Similarly, [^201^Tl]Tl(III)-DOTA appeared relatively stable in RPMl-1640 medium, with 84 ± 2% remaining after 1 h incubation, decreasing to 20 ± 2% at 144 h. The complex was more stable in ammonium acetate buffer with 68 ± 6% of the complex remaining after 144 h incubation (Fig. 4).

## 4 Discussion

lt was confirmed for the first time using the isolated DNA plasmid method that [^201^Tl](III) causes DNA damage; this method has previously been used by ourselves and others to investigate other AE-emitting radionuclides [20,35–44].

ln order to develop bioconjugates of ^201^T1 to evaluate their potential use in MRT, a platform for stable chelation of thallium must first be established. To date, none of the conventional radiometal chelators widely used in nuclear medicine has been adequately evaluated for chelation of radiothallium. Thallium is most stable under ambient conditions in oxidation states (I) and (III). ln oxidation state (I), thallium is known to be strongly hydrated and behaves biologically much like the heavier alkali metals; for example, like potassium, it is a substrate for the sodium-potassium ATPase pump. Moreover, its electronic structure features a sterically active lone pair of electrons. With these properties, it is hard to conceive a likely kinetically stable thallium(I) chelate complex. On the other hand, thallium(III) is electronically analogous to in- dium(III) for which a range of highly stable chelates is known with well-established uses in nuclear medicine. Based on these considerations, thallium(III) would appear to be the more attractive option for developing a suitable chelation system. A prerequisite for developing such a platform is to find an efficient and convenient method to oxidise thallium(I) chloride, the form in which ^201^T1 is manufactured and supplied, to thallium(III). Such a method would need to be sufficienT1y mild to be used in the context of labelling sensitive biomolecules. The T1(I)/T1(III) redox couple has a standard redox potential of +0.77 V, suggesting that unless the metal ion can be stabilised by a chelator, it may be reduced back to Tl(I).

Published methods to oxidise [^201^Tl]Tl(I) to [^201^Tl]Tl(III) included ozone, hydrogen peroxide, HCl or a combination of oxidising agents and high temperatures (95 °C) [32,45,46]. ln our hands, using iT1C-SG plates and acetone as an effective and reliable method to distinguish T1 (I) from T1(III) [46], published oxidation methods (methods 1–4 here) did not always prove successful when evaluated. For example, in method 2, the conversion yield was between 3 and 12%. Although oxidation method 1 was reproducible, heating at 95 °C is not biocompatible (Table 2). Oxidation methods 3–5 used ozone or oxygen as oxidants and avoided the need for high temperatures. Comparing conversion yields obtained from either method 2 or 3 showed the importance of hydrogen peroxide for the oxidation using ozone, although this appeared less important for oxygen (method 5), despite the decrease in oxidation potential from +2.07 V to +1.78 V [47]. Although the solution could be neutralised, this would dilute the radionuclide and increase the complexity of the labelling procedure. Equally, the practical set-up of bubbling oxygen through a reaction vessel using a large cylinder of compressed oxygen adds undesirable complexity and hazard (Table 2). Therefore, alternative, safer methods of oxidation were investigated.

A range of biocompatible oxidising agents has been available for many years, developed for the purpose of radiolabelling biomolecules with radioiodine. Chloramine-T, first used by Greenwood etal. in the 1960s [48], is still popular in this field. In our experiments with oxidation of ^201^T1, we found that conversion yields for chloramine-T were 99%, even at the low amounts previously used to synthesise [^123^I]diiodotyrosyl-salmon calcitonin (0.1 mg) [49]. Although chloramine-T is relatively biocompatible, it is known to cause protein damage in some cases [34], and its presence could lead to misleading stability results or damaged cells during *in vitro* uptake and stability studies described below. It should, therefore, ideally be quenched or removed from the reaction solution prior to introducing the biomolecules. This step is not always simple due to its water solubility. Chloramine-T also has an oxidation potential of + 1.14 V under acidic conditions, so marginally lower than that of oxygen. [50]

We therefore evaluated a range of solid-phase oxidants that could easily be removed after oxidation is complete. Iodo-beads (method 7), for example, consist of a chloramine-T analogue covalenT1y bound to a solid polystyrene bead, allowing the supernatant containing [^201^Tl]Tl (III) to be easily be removed from the vessel; this is also an advantage when using iodogen or TCCA (methods 8–9; Table 2). All three oxidants gave a good conversion yield (99%) from [^201^Tl]Tl(I) to [^201^Tl]Tl(III). An extra advantage of iodogen and TCCA over chloramine T is their solubility in organic solvents and low solubility in water; this enables precoated tubes to be created with the volatile solvent evaporating during the process. Additionally, TCCA has an oxidation potential of +4.84 V which is far higher than ozone and oxygen. [51] Methods 7–9, using iodo-beads, TCCA or iodogen, are thus excellent oxidation methods to convert [^201^Tl]Tl(I) to [^201^Tl]Tl(III) for future MRT using thallium-201. However, in the absence of acid, no conversion to [^201^Tl]Tl(III) was observed. For all the oxidising agents, using 0.1 M HCl leads to a mixture of the starting material and product whereas using 0.5 M HCl leads to the majority of product formation.

Chelation studies showed that while all chelators rapidly and efficienT1y complexed thallium(III), both [^201^Tl]Tl(III)-EDTA and [^201^Tl]Tl (III)-DTPA were unstable in serum, whereas the macrocyclic chelator DOTAperformed better (Fig. 4). As DTPAand EDTAare both acyclic chelators, with 6 and 8 donor atoms, respectively, this instability is likely due to low free energy barriers to conformational changes required to dissociate. The complexes are thermodynamically favourable and quick to form but not kinetically stable. These results conflict with claims in previous studies that [^201^Tl]Tl(III)-DTPA-HlgG, is stable in human serum for more than 24 h [45]. [^201^Tl]Tl(III)-DOTA, on the other hand required longer for the complex to initially form than [^201^Tl]Tl(III)-EDTA and [^201^Tl]Tl(III)-DTPA. The crystal structure of the complex showed the thallium ion direcT1y coordinated to all eight donor atoms in a twisted square antiprismatic coordination and previous work has indicated that DOTA does indeed enable more stable chelation of [^201^Tl]Tl(III) than DTPA, at least *in vitro* [32,52]. A crystal structure of [^nat^Tl]Tl(III)-DOTA obtained by Fodor *et al.* shows the metal sitting above the plane of the cyclen ring [52]. As such, DOTA looks a more promising chelator of [^201^Tl]Tl(III) for MRT than DTPA or EDTA, but it still not an ideal candidate, unless perhaps for a small targeting molecule with a fast biological half-life.

## 5 Conclusion

We have described simple, convenient and mild reactions, using iodo-beads, TCCA or iodogen, to convert DNA-damaging thallium-201 from T1(I) to T1(III), and evaluated a range of conventional chelators for their potential to serve as bifunctional chelators for thallium(III). EDTA and DTPA have inadequate stability for use in bioconjugates for MRT. DOTA shows greater kinetic stability which may suffice for some applications but will unlikely meet the need for a generally applicable thallium bifunctional chelator. This justifies further research into alternative chelators for [^201^Tl]Tl(III).

## Figures and Tables

**Table 1:** Summary of the decay properties of selected Auger electron (AE)-emitting radionuclides: half-life, and average number and energy of AEs per decay. Adapted from Buchegger et al. [1].

lsotope	Physical half-life	AEs per decay	AE energy per decay (keV)
Thallium-201	73 h	36.9	15.3
Gallium-67	78 h	4.7	6.3
lndium-111	67 h	14.7	6.8
lodine-123	13h	24.9	7.4
lodine-125	59.4 d	24.9	12.1

**Table 2:** Conversion yields of [^201^Tl]Tl(I) to [^201^Tl]Tl(III) using oxidation methods 1–9. Values are average ± standard deviation (n = 3). Also shown are characteristics of the nine oxidation methods in terms of simple set-up, ability to remove the oxidising reagent after the reaction, and whether the oxidation process is compatible with radiolabelling biomolecules such as antibodies

Oxidation method	Conversion yield	Simple set-up	Oxidant removed	Biomolecule compatible
1: HCl (6 M), H_2_O_2_, and 95 °C	98 ± 2%	Yes	No	No
2A: HCl (2 M) and ozone	3 ± 2%	No	Yes	No
2B: HCl (6 M) and ozone	12 ± 3%	No	Yes	No
3: HCl (6 M), H_2_O_2_ and ozone	95 ± 5%	No	No	No
4: HCl (2 M), H_2_O_2_ and oxygen	99 ± 1%	No	No	No
5: HCl (2 M) and oxygen	94 ± 6%	No	Yes	No
6A: Chloramine-T (0.5 M HCl)	99 ± 1%	Yes	No	Yes
6B: Chloramine-T (0.1 M HCl)	69 ± 3%	Yes	No	Yes
7A: Iodo-bead (0.5 M HCl)	99 ± 1%	Yes	Yes	Yes
7B: Iodo-bead (0.1 M HCl)	62 ± 8%	Yes	Yes	Yes
8A: Trichloroisocyanuric acid (0.5 M HCl)	99 ± 1%	Yes	Yes	Yes
8B: Trichloroisocyanuric acid (0.1 M HCl)	96 ± 2%	Yes	Yes	Yes
9A: Iodogen (0.5 M HCl)	99 ± 1%	Yes	Yes	Yes
9B: Iodogen (0.1 M HCl)	95 ± 3%	Yes	Yes	Yes
